# Data showing proliferation and differentiation of intestinal epithelial cells under targeted depletion of Notch ligands in mouse intestine

**DOI:** 10.1016/j.dib.2016.12.045

**Published:** 2016-12-29

**Authors:** Toru Nakata, Hiromichi Shimizu, Sayaka Nagata, Go Ito, Satoru Fujii, Kohei Suzuki, Ami Kawamoto, Fumiaki Ishibashi, Reiko Kuno, Sho Anzai, Tatsuro Murano, Tomohiro Mizutani, Shigeru Oshima, Kiichiro Tsuchiya, Tetsuya Nakamura, Katsuto Hozumi, Mamoru Watanabe, Ryuichi Okamoto

**Affiliations:** a Department of Gastroenterology and Hepatology, Graduate School, Tokyo Medical and Dental University, Tokyo, Japan; b Department of Medicine, University of California, San Francisco, San Francisco, CA, USA; c Institute of Clinical Molecular Biology, Christian-Albrechts-University Kiel, D-24105 Kiel, Germany; d Department of Advanced Therapeutics in GI Diseases, Tokyo Medical and Dental University, Tokyo, Japan; e Department of Immunology, Tokai University School of Medicine, Isehara, Japan; f Center for Stem Cell and Regenerative Medicine, Tokyo Medical and Dental University, Tokyo, Japan

## Abstract

The data on the immunohistochemical analysis of conditional Notch ligand knockout mice is presented. Targeted deletion of *Jag1, Dll1, Dll4,* or *Dll1* plus *Dll4* in Lgr5^+ve^ cells was induced by a Cre-mediated gene recombination, and differentiation or proliferation of the intestinal epithelial cells was examined by immunohistochemistry. These data are the extension of the data presented and discussed in the paper entitled “Indispensable role of non-canonical Notch signaling in the proliferation of Apc-deficient intestinal tumors“ (Nakata et al., Submitted for publication) [1].

**Specifications Table** *[please fill in right-hand column of the table below]*

**Table:** 

Subject area	*Biology*
More specific subject area	*Mice intestinal epithelial cell differentiation and proliferation*
Type of data	*Figures*
How data was acquired	*Histology –BZ-X700 (Keyence)*
Data format	*Analyzed*
Experimental factors	*Mouse intestinal tissue*
Experimental features	*Antibody staining documented by histology*
Data source location	*Tokyo Japan*
Data accessibility	*Data is with this article*

**Value of the data**

• Data presented displays the outcome of *Dll1, Dll4*, and *Jag1* depletion in Lgr5^+ve^ cells of the mouse intestine.
• These data serve as a benchmark for future research work regarding the role of Notch ligands in Lgr5^+ve^ cell-dependent intestinal epithelial cell homeostasis.
• The data is valuable for future research works focused on the functional relevance of Notch signaling in intestinal epithelial cell differentiation and proliferation.

## 1 Data

**Fig. 1:**
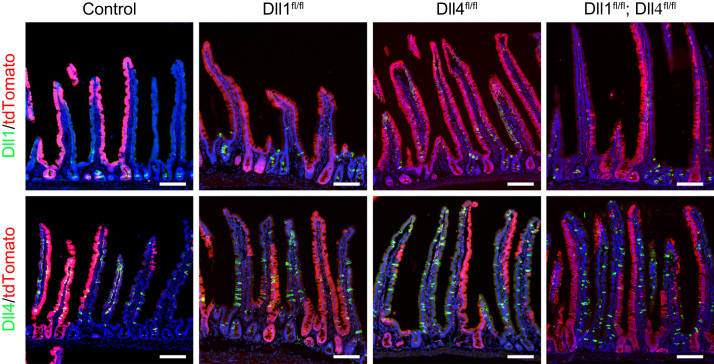
Expression of Dll1 or Dll4 in LGR5-EGFP-ires-CreERT; ROSA26-tdTomato; Dll1^fl/fl^ (Dll1^fl/fl^)mice, LGR5-EGFP-ires-CreERT; ROSA26-tdTomato; Dll4^fl/fl^ (Dll4^fl/fl^)mice, and LGR5-EGFP-ires-CreERT; ROSA26-tdTomato; Dll1^fl/fl^; Dll4^fl/fl^ (Dll1^fl/fl^; Dll4^fl/fl^)mice. Small intestinal tissues of Dll1^fl/fl^ mice , Dll4^fl/fl^ mice , and Dll1^fl/fl^; Dll4^fl/fl^ mice were collected at day 15 after tamoxifen (TX) induction for 5 consecutive days (Days 1–5). LGR5-EGFP-ires-CreERT; ROSA26-tdTomato mice served as control (Control). Analysis of Dll1 (green, upper series) and Dll4 (green, lower series) expression by immunohistochemistry is shown. Red signals indicate tdTomato^+ve^ cells. Scale bar, 100 μm.

**Fig. 2:**
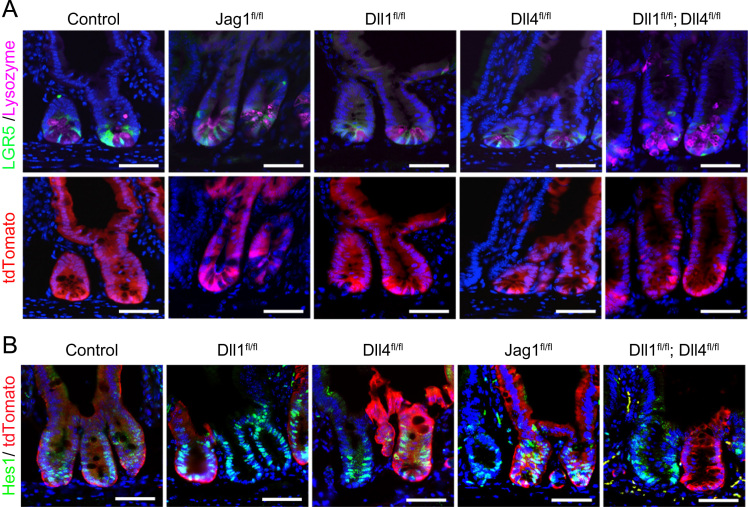
Stem cell niche structure and Hes1 expression in Jag1^fl/fl^ mice, Dll1^fl/fl^ mice, Dll4^fl/fl^ mice, and Dll1^fl/fl^; Dll4^fl/fl^mice. Small intestinal tissues of Jag1^fl/fl^ mice, Dll1^fl/fl^ mice, Dll4^fl/fl^ mice, and Dll1^fl/fl^; Dll4^fl/fl^ mice were collected at day 15 after TX induction for 5 consecutive days (Days 1–5). (A) The number and distribution of LGR5^+ve^ cells (green, upper panel) and Lysozyme^+ve^ cells (red, upper panel) in tdTomato^+ve^ crypt-villus units (red, lower panel) were analyzed by immunostaining. Scale bar, 50 μm. (B) Expression of Hes1 (green) in the small intestinal crypts was analyzed by immunostaining. Red signals indicate tdTomato^+ve^ cells. Scale bar, 50 μm.

**Fig. 3:**
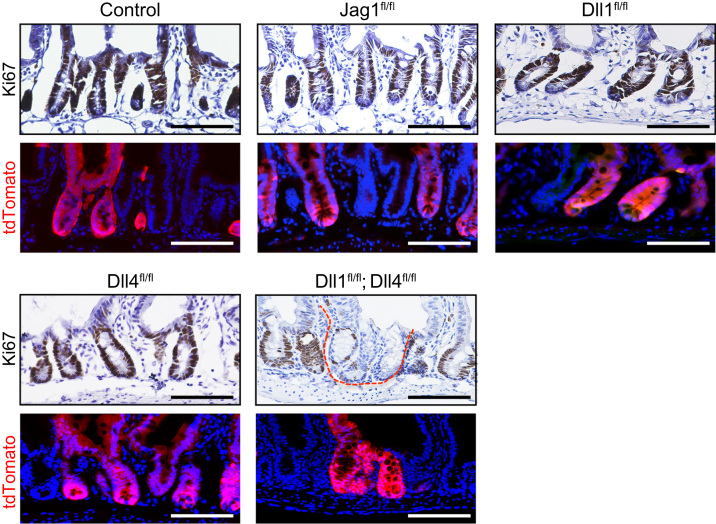
Expression of Ki67 in the small intestinal crypts of Jag1^fl/fl^ mice, Dll1^fl/fl^ mice, Dll4^fl/fl^ mice, and Dll1^fl/fl^; Dll4^fl/fl^ mice. Small intestinal tissues of the designated mice were collected at day 15 after TX induction for 5 consecutive days (Day 1–5). Immunostaining of Ki67 (brown, upper panel) in the tdTomato^+ve^ crypts (red, lower panel) is shown. Data of the lower panel was acquired from the adjacent section of the upper panel. Scale bar, 100 μm.

**Fig. 4:**
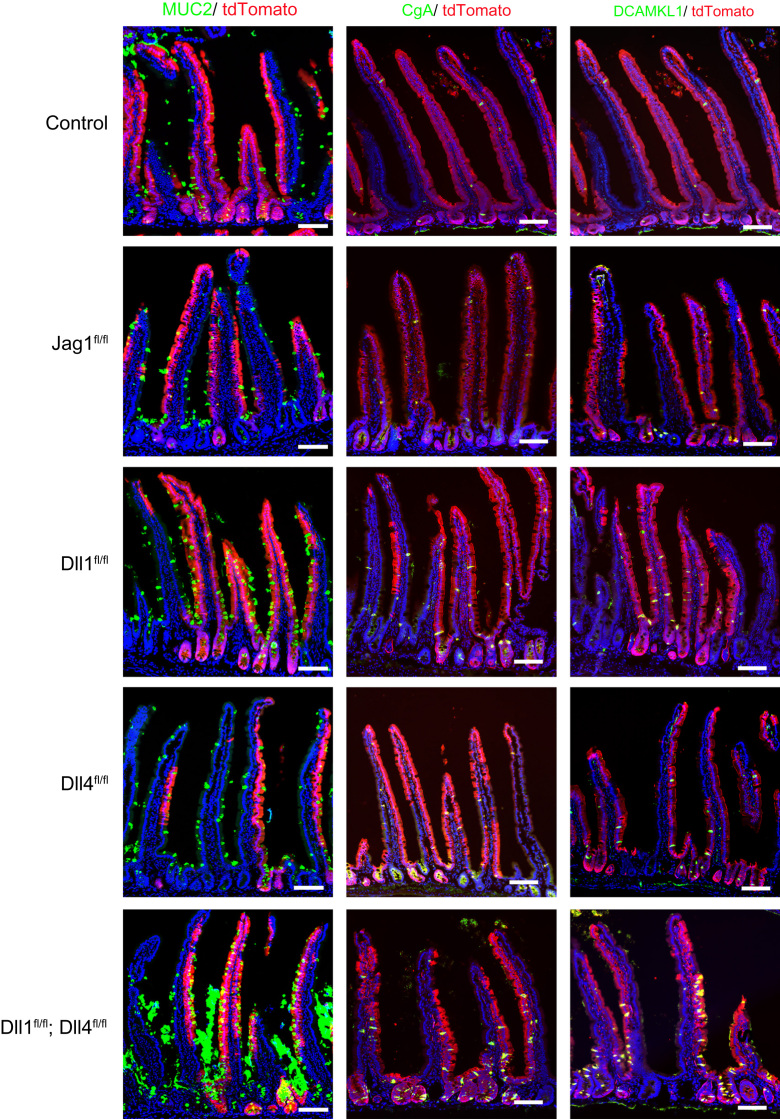
Expression of secretory lineage cell-specific markers in Jag1^fl/fl^ mice, Dll1^fl/fl^ mice, Dll4^fl/fl^ mice, and Dll1^fl/fl^; Dll4^fl/fl^ mice. Small intestinal tissues of the designated mice were collected at day 15 after TX induction for 5 consecutive days (Day 1–5). The number and distribution of goblet cells, enteroendocrine cells, and tuft cells were analyzed by immunostaining of Muc2, CgA, and DCAMKL1 (green) in tdTomato^+ve^ crypt-villus units (red), respectively. Scale bar, 100 μm.

The immunohistochemistry data show the proliferation and differentiation of mouse intestinal epithelial cells under the targeted deletion of *Jag1, Dll1, Dll4*, or *Dll1* plus *Dll4* genes in LGR5^+ve^ cells (Fig. 1, Fig. 2, Fig. 3, Fig. 4).

## 2 Experimental design, materials, and methods

### 2.1 Mice

All the animal experiments were approved by the Animal Welfare Committee of Tokyo Medical and Dental University (Approval no. 016326A). All animal procedures were carried out in compliance with the institutional standards for use of laboratory animals. *Lgr5-EGFP-ires-CreERT2* mice (Stock No. 008875) and *ROSA26-tdTomato* mice (Stock No. 007909) were purchased from The Jackson Laboratory (Bar Harbor, Maine, USA). *Jag1-floxed* (*Jag1*^*fl/fl*^) mice [2], *Dll1-floxed* (Dll1^fl/fl^) mice [3] and *Dll4-floxed* (Dll4^fl/fl^) mice [4] have been previously described. These mice were housed in the animal facility of Tokyo Medical and Dental University, and crossed to generate *LGR5-EGFP-ires-CreERT2; ROSA26-tdTomato; Jag1*^*fl/fl*^ *(Jag1*^*fl/fl*^) mice, *LGR5-EGFP-ires-CreERT2 ROSA26-tdTomato; Dll1*^*fl/fl*^ *(Dll1*^*fl/fl*^) mice, *LGR5-EGFP-ires-CreERT2; ROSA26-tdTomato; Dll4*^*fl/fl*^ (*Dll4*^*fl/fl*^) mice, and *LGR5-EGFP-ires-CreERT2; ROSA26-tdTomato; Dll1*^*fl/fl*^; *Dll4*^*fl/f*^ (*Dll1*^*fl/fl*^; *Dll4*^*fl/fl*^) mice. *LGR5-EGFP-ires-CreERT2; ROSA26-tdTomato* mice served as the control (Control). Cre-mediated gene recombination was induced by intraperitoneal injection of tamoxifen (TX, 2 mg/body) for 5 consecutive days, as previously described [1] [5].

### 2.2 Antibodies

The primary antibodies used are as follows: anti-Dll1 (1:500, AF5026, R&D systems, Minneapolis, USA), anti-Dll4 (1:500, AF1389, R&D systems, Minneapolis, USA), anti-RFP (1:500, PM005, MBL, Nagoya, Japan), anti-tdTomato (1:500, AB8181-20, SIGEN, Cantanhede, Portugal), anti-Hes1 (1:80000, kindly provided by T. Sudo, Toray, Kanagawa, Japan) [6], anti-Ki67 (1:50, TEC-3, DAKO, Glostrup, Denmark), anti-MUC2 (1:100, SantaCruz Biotechnology, Texas, USA), anti-CgA (SP-1, Diasorin, Saluggia, Italy), anti-DCAMKL1 (1:100, AP7219B, Abgent, San Diego, USA), and anti-Lysozyme (1:1500, EC3.2.1.17, DAKO, Glostrup, Denmark).

### 2.3 Immunohistochemistry of mouse intestinal tissue samples

Immunohistochemistry of mouse intestinal tissues was performed as previously described [5], [7]. Sections (8 μm) were prepared for the analysis. Antigen retrieval in citrate buffer was required for staining Dll1, Dll4, CgA and Hes1. Tyramide signal amplification was used for the immunofluorescent detection of Dll1, Dll4, CgA and Hes1. Stainings were visualized by the standard Avidin-biotin complex (ABC) method, or by secondary antibodies and tyramide substrates conjugated with Alexa-594 or Alexa-488 (Molecular Probes, California, USA). Tissues were counterstained by 4׳,6-diamidino-2-phenylindole (DAPI) or by hematoxylin. Data were collected using an epifluorecent microscope (BZ-X700, KEYENCE, Tokyo, Japan).

## Funding

This study was supported by MEXT/JSPS KAKENHI grant number 25293170 (to RO, KT and TN), grant number 23102003 (to RO and TN), grant number 15K15286 (to RO), grant number 16H05284 (to RO) and grant number 226221307 (to KT, TN and MW); the Research Center Network Program for Realization of Regenerative Medicine from Japan Agency for Medical Research and Development, AMED, grant number 16bm0304001h0004 (to RO, TN, KT and MW); the Practical Research Project for Rare / Intractable Diseases from Japan Agency for Medical Research and Development, AMED, grant number ​16ek0109188h0001 (to RO).

## Figures and Tables

**Fig. 5:**
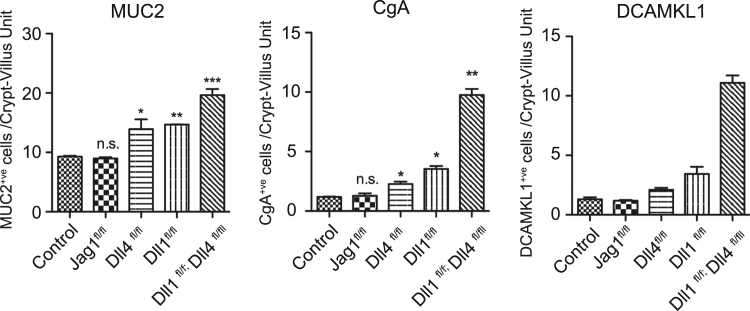
Quantification of Muc2^+ve^ Cells, CgA^+ve^ cells, and DCAMKL1^+ve^ cells in tdTomato^+ve^ crypt-villus units by immunostaining of small intestinal tissues of the designated genotype at day 15 from TX induction. Representative staining for Muc2, CgA, and DCAMKL1 is shown in Fig. 4. Data shows mean±SEM of triplicate experiments (n=3). * indicates P<0.05, ** indicates P<0.01, *** indicates P<0.001 as determined by Student’ s t-test. n.s. indicates not significant.
